# Unlocking fresh perspectives: molecular breakthroughs in pediatric acute myeloid leukemia classification and prognosis

**DOI:** 10.1002/mco2.750

**Published:** 2024-09-15

**Authors:** Yu Tao, Li Wei, Hua You

**Affiliations:** ^1^ Laboratory for Excellence in Systems Biomedicine of Pediatric Oncology Department of Pediatric Hematology and Oncology Chongqing Key Laboratory of Pediatric Metabolism and Inflammatory Diseases Ministry of Education Key Laboratory of Child Development and Disorders, National Clinical Research Center for Child Health and Disorders Children's Hospital of Chongqing Medical University Chongqing China; ^2^ NHC Key Laboratory of Birth Defects and Reproductive Health Chongqing Population and Family Planning Science and Technology Research Institute Chongqing China

**Keywords:** clinical outcomes, molecular categories, pediatric acute myeloid leukemia, risk stratification

1

Recently, a novel transcriptome and genome profiling study published in the *Journal of Nature Genetics*, expanded the prognosis‐related molecular classification coverage of pediatric acute myeloid leukemia (pAML) from 68.5% (as defined by the WHO 5th edition) to 91.4%.^1^ This framework was strongly associated with clinical outcomes, potentially shaping future classifications and treatment of pediatric AML.

Differences in molecular profiles between pediatric and adult AML restrict the use of risk stratification tools designed for adults when applied to pediatric patients. For instance, while the TP53 mutation, present in about 8% of adult AML cases and linked to poor outcomes, is emphasized in the European LeukemiaNet (ELN) 2022 guidelines,^2^ it is infrequently observed in pediatric AML. Furthermore, numerous driver alterations that are specific to pediatric cases are not adequately represented in the existing classification schemas, and risk stratification for pediatric AML is still evolving. This prompted Umeda and colleagues to comprehensively explore the increasingly intricate genomic landscape within the framework of the latest hematological malignancy classification systems and to create a categorization system uniquely designed for pediatric AML.

In their study, RNA sequencing (RNA‐seq) data of 887 pAML patients were assessed, complemented by DNA sequencing data, allowing for a comprehensive examination of genetic features, including internal or partial tandem duplications (ITD/PTD), copy‐number variations, single nucleotide mutations, fusions, and insertions and deletions (indels). It was revealed that while WHO 5th identified 68.5% of pAML cases with specified genetic alterations, the new pAML classification system, which incorporates 12 additional molecular categories, captures 91.4% of cases. The discovery of these new major entities such as UBTF tandem duplications, GLIS family rearrangements, and BCL11B structural variants and outlier expression will lead to greater attention and analysis of these patients’ biological and clinical features.

Further clinicopathological association analysis revealed that pAML morphological features are defined by the identified driver alterations and developmental stages. Given that numerous category‐defining alterations are either cytogenetically obscure or involve somatic mutations, this underscores the necessity for sequencing techniques to achieve precise molecular diagnosis of pAML. As for gene expression, molecular categories with favorable prognosis (such as CBFB::MYH11, CEBPA, RUNX1::RUNX1T1) typically exhibited high granulocyte–monocyte progenitor scores. Conversely, KMT2Ar, associated with poor prognosis, had low stemness‐related scores and variable differentiation‐related scores. The differences in the aforementioned prognosis or drug–response‐related patterns reflect that molecular categories are associated with unique pathophysiological characteristics. Considering inter‐categorical similarities resulted in the formation of extensive clusters, encompassing AMKL/AEL, CBF leukemias, immature AML, CEBPA, and two clusters characterized by HOXA and HOXB gene expression. Specifically, the HOXA and HOXB groups demonstrate notable disparities in the expression of stemness‐related, monocyte, or signaling‐related genes, as well as in mutational patterns. Molecular categories featuring HOXB signatures were closely correlated with FLT3‐ITD and WT1 mutations, whereas categories with HOXA signatures were linked to KRAS mutations. This finding provides a theoretical basis for the common biological mechanisms and potential personalized therapeutics for the same cluster.

Among 887 pAML patients, 76 cases remained unclassified. Twenty‐one had known driver alterations, nine had no detectable pathogenic alterations, and the rest showed at least one pathogenic alteration involving genes like myelodysplasia‐related genes, ETV6, RUNX1, and TP53, along with complex karyotypes, without defining a specific subtype. Further molecular data collection and functional experiments are required to properly classify these patients.

At this point, we have gained a comprehensive understanding of the mutational and expression characteristics of these 23 molecular categories. However, are these molecular categories, especially those newly defined molecular categories, associated with clinical outcomes? Data from the AAML1031 trial confirmed associations between these categories, age at diagnosis, FLT3‐ITD involvement, and minimal residual disease (MRD) positivity.^3^ While the prognosis of the majority of the well‐established major categories remained consistent with previous findings, a strong correlation between new molecular categories and outcomes was observed. For instance, PICALM::MLLT10, UBTF, and KAT6Ar were identified as high‐risk group, while CBFB‐GDXY insertions were categorized as low risk. They utilized recursive partitioning models to analyze event‐free survival time of molecular categories and KMT2Ar fusion partners, revealing three distinct prognostic groups. Cox proportional hazards modeling showed that identified risk groups and MRD positivity were independent prognostic factors. This resulted in the creation of a predictive framework that combines molecular categories and MRD positivity, leading to six risk strata with detailed outcome forecasts. Validation using the AML08 trial cohort confirmed the prognostic significance.^4^ Additionally, the predictive capability of this prognostic framework was found to be on par with or better than several risk stratification methods currently employed in clinical trials for pediatric AML or the ELN 2022 guidelines for adult AML.^2^

Overall, the pAML‐focused categorization presented by Umeda et al. proposed 23 mutually exclusive molecular categories, with 12 new molecular categories not currently defined by WHO 5th (Figure 1). This comprehensive molecular diagnostic and prognostic framework might provide the foundation for future risk classification of pAML and the refinement of treatment strategies. Moreover, based on subsequent systematic analyses of biological characterization, gene expression signatures, superfamily identification, and clinical associations, the comprehensive dataset generated by this study will be an invaluable asset for researchers working in the field of pAML.

**FIGURE 1:**
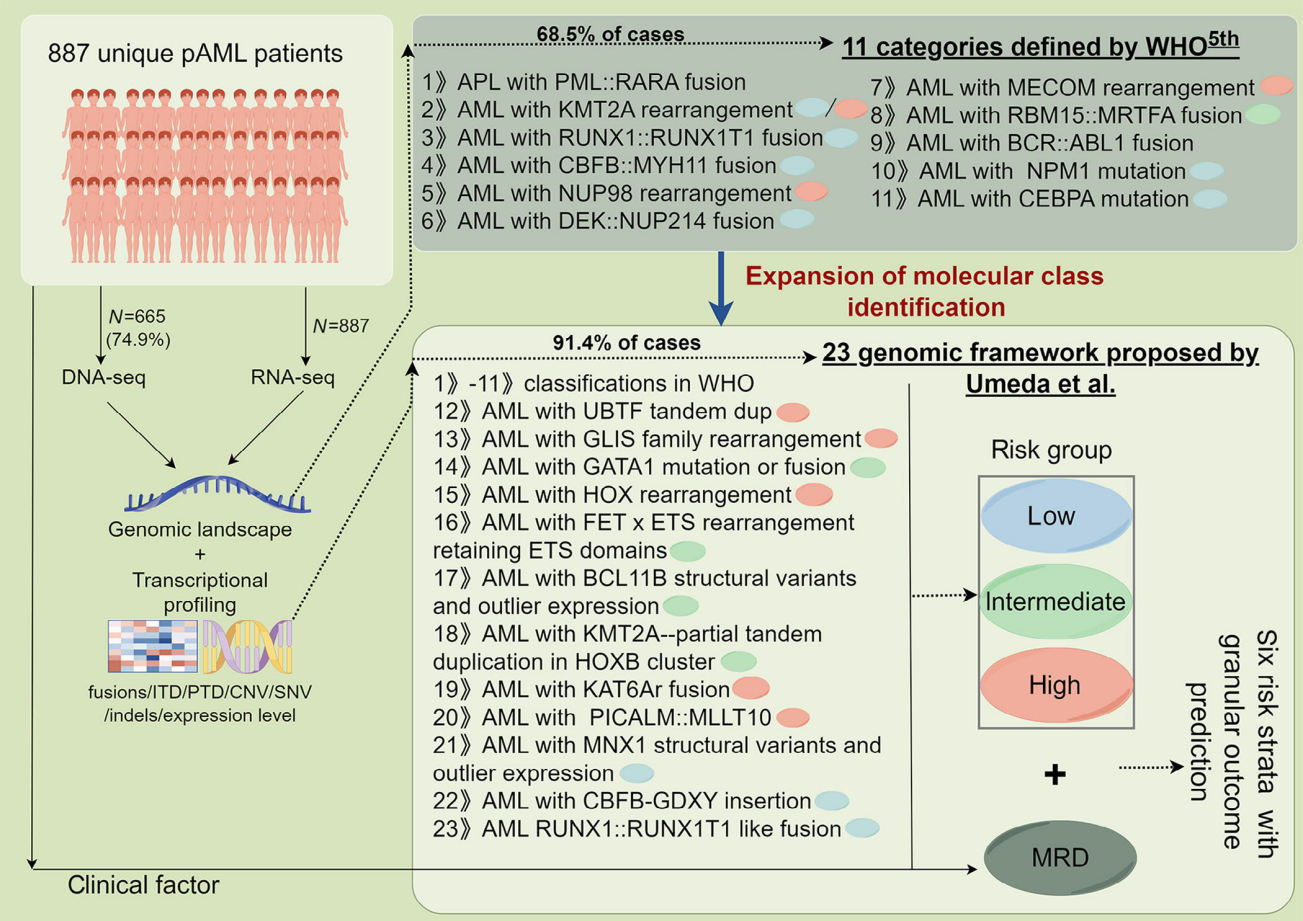
Landscape of the newly proposed 23 molecular categories and comparison with the WHO 5th classifications for pediatric AMLs (created in www.figdraw.com).

Although the encouraging results of this work provides a strong theoretical foundation for understanding the heterogeneity of pAML and for future risk stratification and clinical decision‐making, there remain some inevitable challenges in translating these findings into clinical applications. First, the strength of this framework depends heavily on RNA‐seq data analysis for canonical and cryptic genetic calling. It is essential to develop user‐friendly pipelines in the future, given the global lack of universal access to clinical sequencing and the significant expertise required for these molecular analyses. Second, although the framework proposed in this article, when combined with MRD, shows excellent prognostic prediction for pAML patients, it is important to also consider the unique clinical features of pediatric AML. For instance, it is widely acknowledged that pAML are at a greater risk of central nervous system (CNS) involvement and/or CNS relapse compared with adults, a factor that was not considered in the development of the system presented in this article. Moreover, a thorough comparison between the Chinese and Western AML cohorts has already revealed a notably different genomic alteration profile, which was not covered in this study.^5^

Since this study is retrospective, we are looking forward to prospective experiments to enhance clinical applicability of the framework. Although high‐risk patients identified in this study might benefit from HSCT, previous research has shown that certain high‐risk groups, like those with FUS::ERG, may not benefit from this treatment. Additionally, there is a substantial proportion of low‐ and intermediate‐risk patients who are in need of more effective and personalized treatment options. Consequently, in the contemporary high‐resolution genomic era, future risk stratification should increasingly focus on identifying targetable lesions to facilitate the integration of molecularly targeted therapies for pAML.

## AUTHOR CONTRIBUTIONS

H. Y. designed the research. Y. T. and L. W. wrote and revised the manuscript. All authors have read and approved the final manuscript.

## CONFLICT OF INTEREST STATEMENT

All the authors declare no conflict of interest.

## ETHICS STATEMENT

Not applicable.

## Data Availability

Not applicable.
